# Repair of finger pulp defects using a free second toe pulp flap anastomosed with the palmar vein

**DOI:** 10.1186/s13018-022-03232-z

**Published:** 2022-07-16

**Authors:** Xiaoli Zhang, Ziyu Wang, Xiaoning Ma, Yanyan Jin, Jingxin Wang, Hang Yu, Tong Li, Xiaolei Xiu

**Affiliations:** 1 Cangzhou Hospital of Integrated Traditional Chinese and Western Medicine, No.31 Huanghe Road, Yunhe District, Cangzhou, Hebei China; 2 North China University of Science and Technology, Tangshan, Hebei China

**Keywords:** Toe pulp flap, Hand injury, Digital pulp defect, Second toe, Transplantation

## Abstract

**Purpose:**

To evaluate the clinical utility of surgical reconstruction of finger pulp defects using a plantar flap derived from the toes, with vascular anastomosis of the toe-finger artery and the plantar-palmar vein of the finger.

**Methods:**

Between April 2018 and November 2020, 29 patients with finger pulp defects underwent treatment via the transplantation of pulp tissue from the second toe, with the plantar vein of the toe and the palmar vein of the finger being anastomosed during this procedure. In addition, an anastomosis of the toe and finger artery and nerve was conducted, with a flap size of 1.0 cm * 0.8 cm–2.3 cm * 4.0 cm being used for such repair. Donor tissue sites were closed without introducing deformities or other complications.

**Results:**

In all patients in the present study, flap tissues survived and did not exhibit evidence of vascular crisis over a mean 16.8-month follow-up period (range 8–24 months). After successful skin flap grafting, they exhibited good elasticity and a soft texture. At three months post-surgery, some patients reported partial recovery of touch sensation in the transplanted tissue, while pain recovery was evident in some patients at 4–6 months post-surgery. No deformities or other complications were observed at the donor site, and the ability of patients to walk normally was not impaired.

**Conclusion:**

The anastomosis of toe plantar flaps with the palmar vein can facilitate the repair of finger pulp injuries without the need to dissect the dorsal vein of the toe, allowing for the suturing of donor tissue sites without causing any deformities or other complications. This surgical approach can easily be conducted with satisfactory clinical outcomes.

## Introduction

While automated approaches are replacing manual labor in many industries, these machines have the potential to cause serious and irreversible damage to hands and other soft tissues. Soft tissue defects of the fingertip are a common form of hand trauma [1], and when accompanied by the exposure to tendon and bone, often require a flap for repair [2]. Hand surgeons must choose a treatment option by considering the condition of the wound, concomitant injuries, and underlying disease status. Treatment method selection is also related to the economic and physical status of patients [2]. In addition, the ideal skin flap has the characteristics of easy acquisition, good ductility, optimal matching with respect to the color and texture of the recipient skin, matching with respect to the size of the defect, a pedestal of similar size to the recipient blood vessel, low damage to the donor site, and reasonable flap size. Aesthetics is an important consideration for most people, and the hands are essential for completing most of the tasks encountered in daily life. As such, ensuring appropriate hand aesthetics and function is vital. Thus, an ideal reconstruction approach would produce satisfactory aesthetic and functional outcomes.

Skin grafts cannot be directly applied to wounds with exposed bones and tendons [3]. Many such approaches have been detailed to date, including the cross-finger flap, the unilateral proper digital artery advancement flap, the reverse digital artery island flap, the reverse dorsal metacarpal flap, and thenar perforator flap approaches. The flaps used in these surgical operations, however, have limited functionality and a poor appearance in many cases, and some require multiple operations such that the flaps are unable to move freely after initial surgical treatment, causing patients to suffer from joint stiffness. However, utilizing free toe flaps can overcome these limitations by meeting both the physical and psychological requirements of injured patients, due to their size, shape, and position can be adjusted as necessary. Free toe pulp flaps are suitable for the reconstruction of a range of hand soft tissue defects. In addition, there is substantial anatomical similarity between the skin of the toe and that of the hand [4], the selection of a foot skin flap can maintain the similarity with the missing tissue and reconstruct hand defects with respect to both appearance and function. Relative to the back of the hand, the skin of the palm is thicker and less moveable [5]. At the final follow-up, all patients were satisfied with the functional and aesthetic appearance, and no patients required additional aesthetic improvement procedures. Hong et al. [6] correlated the clinical outcomes of partial second toe pulp free flaps for acromegaly in young children and showed that all 18 toe medullary flaps survived in 17 patients without any major complications. At follow-up, the flap-covered distal phalanges showed growth consistent with normal development. In conjunction with previous studies, partial second toe pulp free flaps have been shown to be the best option for fingertip reconstruction in young children and adults.

A free second toe flap can be used to repair finger pulp defects, and this flap provides a satisfactory appearance and functional recovery. In this report, we describe a technique for repairing finger pulp defects with a free flap of the second toe without damaging the function of the toe, and we present the corresponding aesthetic and functional results.

## Materials and methods

### General information

A total of 29 patients were included in the present study, including 17 males and 12 females with a mean age of 25.8 years (range 17–56). The causes of injury included 23 cases of machine strangulation, 4 cases of crush injuries and 2 cases of door squeeze-induced injury. The resulting defects included a dorsal defect of the proximal little finger, a degloving injury of the distal index finger pulp, and a defect of the middle finger, and these injuries were all repaired using pulp flaps from the side of the second toe. There were 32 flaps in total, including the index finger 10 cases, the middle finger 10 cases, the ring finger 7 cases and the little finger 5 cases. The flap area used for tissue repair was 1.0 cm * 0.8 cm–2.3 cm * 4.0 cm. The wound sites in all patients were initially covered with artificial skin (Unitrump Bio Company, Jiangsu, China) or oil gauze after primary treatment and infection control, with surgery performed 5–7 days later. Repair was conducted using a second toe flap harvested from the same or contralateral side, with all flaps being transferred as free flaps. The patient details are compiled in Table 1. All patients were followed up for at least 6 months and the occurrence of complications such as infection and necrosis was recorded during the follow-up. Also, at the last follow-up, gait was observed by visual analysis, and pain sensation in the fingers was measured by visual analog scoring (VAS) using the eight-point scale of the RLA (Rancho Los Amigos) Rehabilitation Hospital in California, and the distance between the two points of discriminative sensation of the fingers was measured at the same time. Patients were evaluated for efficacy.

**Table 1:** Patient characteristics

Case	Sex	Age	Affect finger	Pattern of defect	Skin defect size(cm)
1	M	19	Right little finger	Digital pulp defect of little finger	1.0 × 0.8
2	M	50	Left index finger	Digital pulp defect of index finger	1.0 × 0.8
3	F	35	Left middle finger	Digital pulp defect of middle finger	1.5 × 2.5
4	F	43	Left middle finger	A dorsal defect of the proximal middle finger	1.5 × 3
5	F	49	Left little finger	Digital pulp defect of little finger	1.2 × 1.0
6	M	18	Left little finger	Digital pulp defect of little finger	1.0 × 0.8
7	F	47	Left index finger	Digital pulp defect of index finger	1.5 × 2.0
8	M	37	Right ring finger	Digital pulp defect of ring finger	1.5 × 2.0
9	M	21	Right little finger	Digital pulp defect of little finger	1.5 × 2.0
10	F	37	Left middle finger	Digital pulp defect of middle finger	1.5 × 1.0
11	M	17	Left index finger	A dorsal defect of the proximal index finger	1.8 × 2.5
12	M	27	Right middle finger	Digital pulp defect of middle finger	1.5 × 2.0
			Right ring finger	Digital pulp defect of ring finger	1.5 × 1.0
13	M	39	Right middle finger	Digital pulp defect of middle finger	1.3 × 1.0
14	M	36	Right index finger	Digital pulp defect of index finger	1.5 × 2.5
15	M	56	Right index finger	Digital pulp defect of index finger	1.5 × 2.0
16	M	21	Left middle finger	Digital pulp defect of middle finger	1.0 × 0.8
			Left ring finger	Digital pulp defect of ring finger	1.5 × 1.5
17	F	36	Right middle finger	Digital pulp defect of middle finger	1.2 × 1.0
18	M	30	Left index finger	Digital pulp defect of index finger	1.5 × 1.0
19	F	45	Left ring finger	Digital pulp defect of ring finger	1.6 × 2.0
20	M	38	Left index finger	Digital pulp defect of index finger	1.6 × 2.0
21	M	17	Right middle finger	Digital pulp defect of middle finger	1.5 × 1.0
22	F	38	Right index finger	Digital pulp defect of index finger	2.3 × 4.0
23	F	39	Right index finger	Digital pulp defect of index finger	2.0 × 1.0
24	M	54	Right ring finger	Digital pulp defect of ring finger	1.3 × 2.0
25	M	29	Left index finger	Digital pulp defect of index finger	1.5 × 3.0
26	F	38	Left middle finger	Digital pulp defect of middle finger	1.0 × 0.8
			Left ring finger	Digital pulp defect of ring finger	1.0 × 0.8
27	F	52	Right little finger	Digital pulp defect of little finger	1.5 × 2.0
28	M	21	Left ring finger	Digital pulp defect of ring finger	1.5 × 2.0
29	F	50	Right middle finger	Digital pulp defect of middle finger	1.0 × 1.0

### Operative approach

Prior to surgery, the second toe flap was designed for each patient based on the characteristics of the fingertip defects. The contralateral toe was selected for injuries to the index and middle fingers, while the ipsilateral toe was used to repair injuries to the ring and little fingers. The toe and finger surgeries were performed on the arm under block anesthesia. During the operation, the skin was incised at the proximal end of the designed flap along the toe and separated to both sides, exposing and protecting the plantar vein of the second toe. It is generally sufficient that 1–2 veins greater than 0.5 mm in diameter be preserved, cutting them off the proximal end along both sides of the vein to an appropriate length, with the proximal end subsequently being ligated. The tibial nerves and vascular bundles of the second toe were then separated, exposed, cut at appropriate positions, and the proximal ends of these vessels were ligated. After separation of these blood vessels, the trochanter was attached and the skin flap was cut, with careful attention being paid to preserve the adipose tissue inside the flap so as to ensure that the repaired fingertip would exhibit a rounded appearance. The donor site was then sutured, and the tourniquet was loosened to ensure that the toe received an appropriate blood supply. The percutaneous flap was then transplanted to the injured finger region, with the distal and lateral sides of the flap sutured. A “z”-shaped incision was made on the palm side of the repaired finger, and a subcutaneous vein of appropriate caliber was located. The blood vessels and nerves of the dominant side of the finger were then exposed and anastomosed and sutured to those of the toe flap, with the proximal vein of the flap and the palmar vein of the finger being anastomosed. The proximal wound was then sutured, and the tourniquet was loosened and bandaged. After surgery, patients routinely received prophylactic anti-infection, analgesia, and baking lamp irradiation (24 h irradiation for 7 consecutive days, temperature was controlled between 37  ℃ and 40  ℃), and were asked to stay in bed for 7–10 days to promote wound healing, while anticoagulants were added to prevent thrombosis.

### Observation index and evaluation method

All patients were followed up for at least 6 months, and their efficacy was evaluated at the last follow-up visit and complications were recorded. Gait was observed using visual analysis and rated using the eight-point scale of the RLA (Rancho Los Amigos) [7] Rehabilitation Hospital, California, USA; pain sensation in the fingers was measured by VAS [8], and the distance between the two-points discriminative (2-PD) sensation of the fingers (defined as the minimum distance between 2 points of stimuli on skin) [9] was also measured by a Jamar Hands On Evaluation Kit (Sannyi Healthcare Corporation, Guizhou, China).

## Results

In the 29 patients included in the present study cohort, all 32 flaps survived without evidence of arterial or venous crises. The flaps exhibited good postoperative blood circulation, a full shape and satisfactory elasticity. Patients were followed for up to 8–24 months. During this period, the average subjective satisfaction score of patients at the last follow-up visit was 8 (range 5–10). Pain sensation as measured using VAS was partially restored in some patients (17/29; 59%). Most patients had partial tactile recovery of the finger on the 2-PD test (15/29; 52%). All patients returned to their jobs, and none had complications at the donor site, exhibiting normal foot morphology and walking function.

## Typical case overview

### CASE 1

**Fig. 1:**
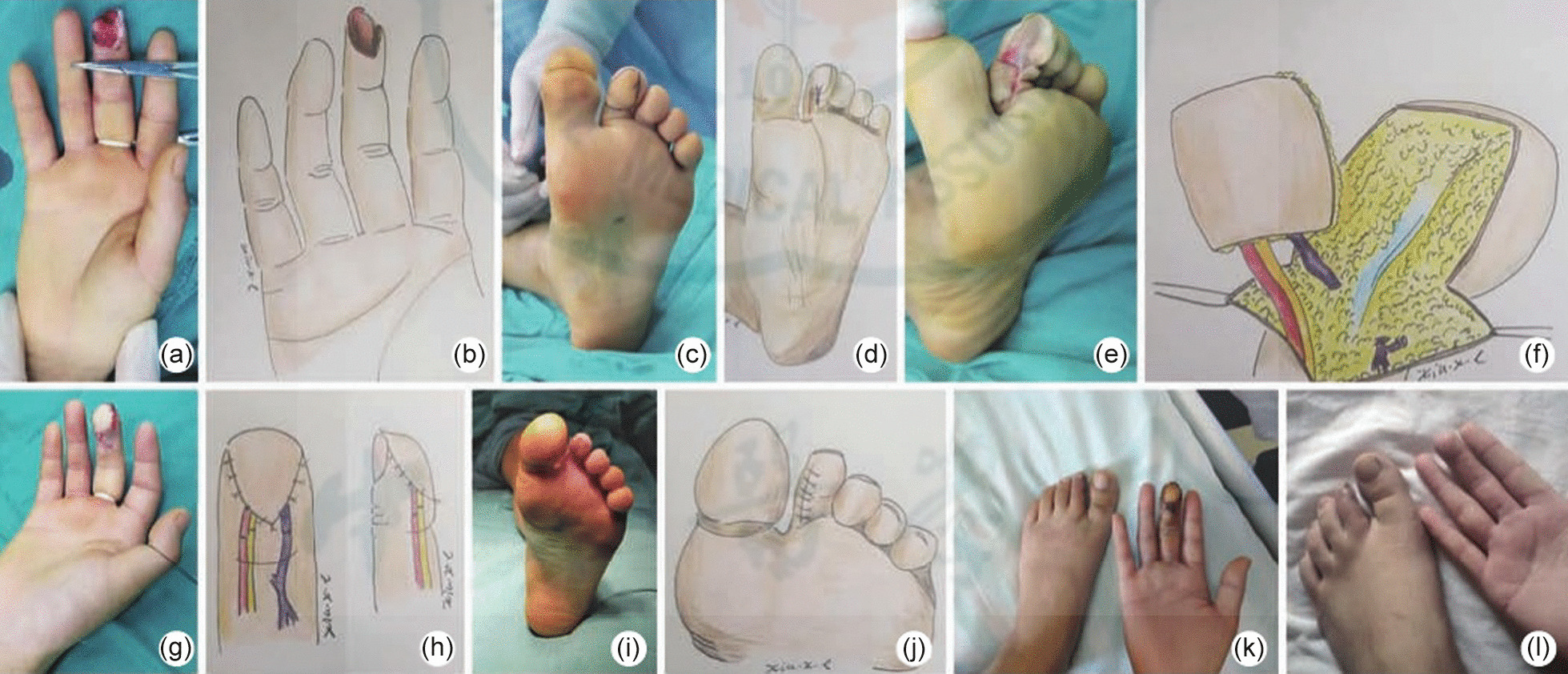
**a** A preoperative image of a middle finger pulp defect of the right hand. **b**. Schematic diagram. **c** Design of the second toe plantar flap in the contralateral foot. **d**. Schematic diagram. **e** Flap collection. **f** Schematic diagram. **g** Recipient site appearance. **h** Schematic diagram. **i** Direct suturing of the donor site. **j** Schematic diagram. **k** Flap survival status. A. Schematic diagram **l** At a 3-month postoperative follow-up, the repaired middle finger and donor sites exhibited a satisfactory appearance

A 36-year-old female patient was admitted to the hospital due to a pulp defect in her right middle finger as a result of a machine-induced injury (Fig. 1 a**–**b). Initially, the wound was covered with artificial skin, and after 7 days the contralateral second toe tibial toe flap was prepared (Fig. 1 c–d). The wound area was 1.2 cm * 1.0 cm, and the flap used to repair this wound was 1.2 cm * 1.0 cm. The wound generated at the donor site was directly sutured. The flap survived the first stage of this procedure (Fig. 1 e–k), and exhibited a satisfactory appearance at 3 months postoperatively (Fig. 1 j).

### CASE 2

**Fig. 2:**
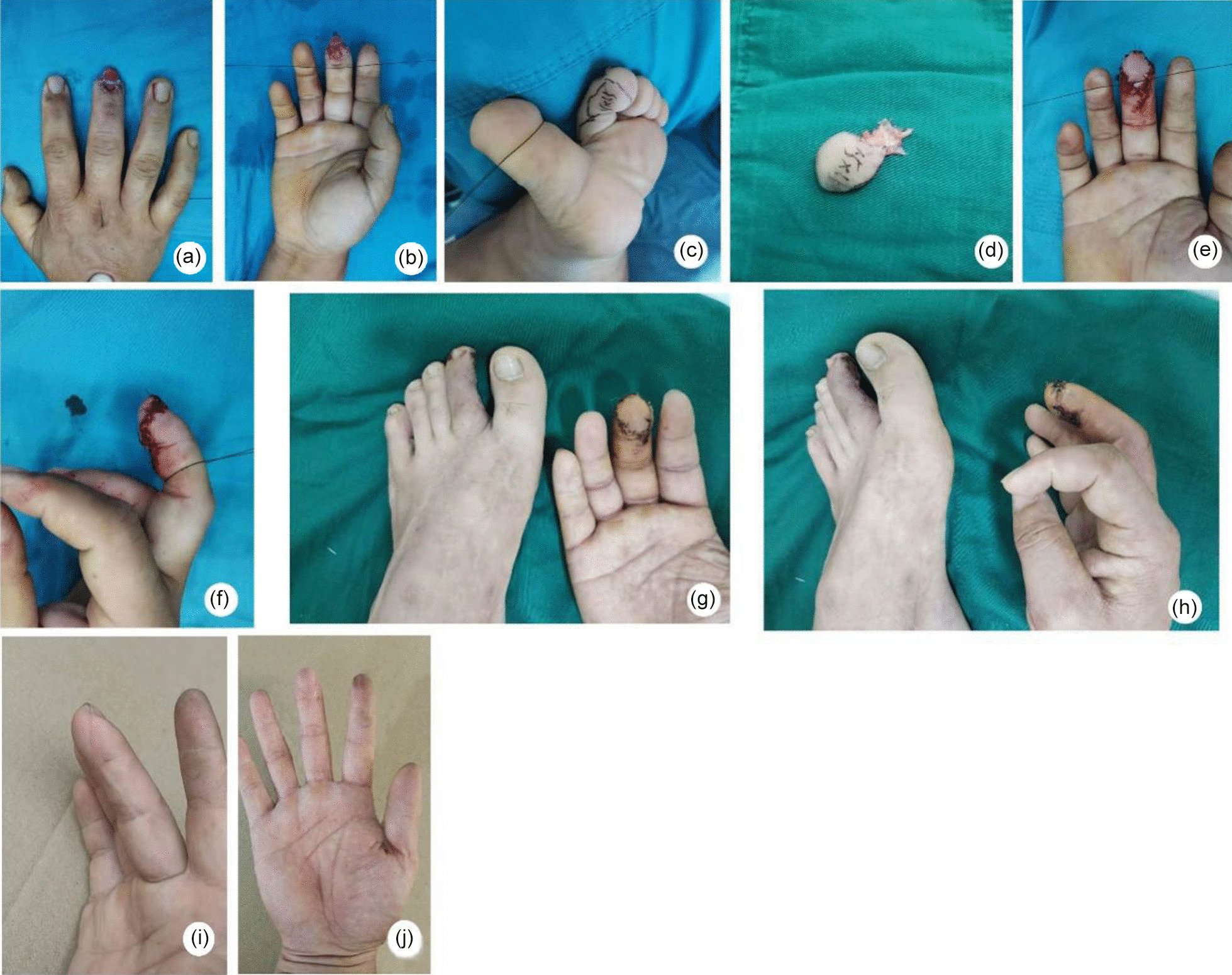
**a–b** A preoperative image of a middle finger pulp defect of the right hand. **c** Design of the second toe plantar flap in the contralateral foot. **d** Flap collection. **e–f** Recipient site appearance. **g–h** 10 days after the operation. **i–j** At 2-year postoperative follow-up

A 39-year-old male patient was admitted to the hospital because of "pain and bleeding for an hour after a trauma to the right finger.” In the first stage of emergency repair, an artificial skin membrane was used under local anesthesia. The wound was covered with artificial skin, and after 7 days the contralateral second toe tibial pulp flap (1.3 cm × 1.0 cm) was used to repair the defect. (Fig. 2 a–d) The flap used to repair this wound was 1.3 cm * 1.0 cm. The wound of the donor site was sutured directly, and the flap survived in the first stage (Fig. 2 e–f). Ten days after the operation (Fig. 2 g–h), 3 months after the operation, the appearance was satisfactory, the feeling of finger pulp recovered well, and the function recovered well (Fig. 2 i–j).

## Discussion

The distal phalanx of the finger must be able to execute fine movements, including holding and pinching, and contains a wide range of tactile receptors and nerve sensory bodies. Finger pulp soft tissue defects are a relatively common form of hand injury, and because these injuries often result in exposure of nerves, tendons, and bone, a flap is generally required to cover the wound site [10]. In the past, the unilateral proper digital artery advancement flap and contralateral hand thenar flap approaches have been used to repair such pulp injuries, but these strategies generally achieve poor sensory recovery and necessitate skin grafting at the donor site, resulting in additional scar formation [11–13] Recent advances in microsurgical technologies have significantly improved small vessel anastomosis reliability, and as such, the toe-toe fibular flap and the second toe tibial flap are often used to repair soft tissue defects in the fingertips. Owing to the similar skin properties of the hands and feet, these flaps are threaded, wear-resistant, and realistic in appearance, such that they are increasingly the first choice for microsurgeons [14, 15]Lee et al. [16] previously utilized 929  s toe flaps to repair 854 cases of finger pulp defects over a 7-year period, including 57 cases with two-finger defects, and 9 cases with three-finger defects. They achieved a flap survival rate of 99.7%. In our study, a total of 32 flaps were transplanted in 29 patients with a 100% flap viability rate, and we had a minimum follow-up of 8 months or more. This is basically consistent with the previous research results.

In this study, a free flap of the second toe was taken and anastomosed to the plantar side of the palmar veins, arteries, and nerves to repair a soft tissue defect of the finger. They have a similar structure and the toe flap can be easily and quickly detached during the procedure without changing the patient's position. Kwon et al. [17] conducted a study on the efficacy of surgical reconstruction of the fingertip with an unmyelinated flap of the second toe and showed that the flap size was 2.39 (range 1.5–5) × 1.29 (range 1–1.8) cm^2^ and all flaps survived. Two patients had venous congestion, one patient had neuroma and two patients had partial necrosis, all of whom recovered with conservative treatment. The mean follow-up period was 5.79 (range 2–18) months, and all patients had no functional impairment. Our study found that if the flap width was within 1.0 ± 0.2 cm, the donor site could be sutured directly without skin grafting, and the resulting incision could be easily concealed, and the final follow-up of our study found no significant abnormalities in foot appearance or motility in this group of patients.

When using the second toe flap in the present study, we found that there were always 2–3 small veins on the plantar side of the toe that converged into a vein that was suitable for anastomosis closer to the proximal end. However, this vein was relatively thin, so caution is essential when separating it, as this process is easier when the vein is filled with blood. Although this operation has many advantages, and a series of results show that patients and surgeons are satisfied with the operation, there are a few disadvantages to this operation. For one, the surgical approach necessary to cut the plantar vein requires access to a high degree of microsurgical technology, as it is a challenging and potentially risky operation. It is important that blood not be removed from the lower extremities too thoroughly during the operation, as it is easier to gauge the diameter and remove the vein when it still contains blood. Second, it is possible that plantar veins may be absent. Intraoperatively, the proximal end of the flap is cut to determine whether these veins are present. When they are absent, a second set of dorsal toe veins should be prepared.

## Data Availability

The datasets are available from the corresponding authors on reasonable request.
